# Analysis of LGR4 Receptor Distribution in Human and Mouse Tissues

**DOI:** 10.1371/journal.pone.0078144

**Published:** 2013-10-21

**Authors:** Jing Yi, Wei Xiong, Xing Gong, Seth Bellister, Lee M. Ellis, Qingyun Liu

**Affiliations:** 1 Brown Foundation Institute of Molecular Medicine and Texas Therapeutics Institute, University of Texas Health Science Center at Houston, Houston, Texas, United States of America; 2 Department of Surgical Oncology, The University of Texas M.D. Anderson Cancer Center, Houston, Texas, United States of America; Sanford Burnham Medical Research Institute, United States of America

## Abstract

LGR4 is an R-spondin receptor with strong positive effect on Wnt signaling. It plays a critical role in development as its ablation in the mouse led to total embryonic/neonatal lethality with profound defects in multiple organs. Haplotype insufficiency of LGR4 in human was associated with several diseases, including increased risk of squamous cell carcinoma of the skin, reduced birth weights, electrolyte imbalance, and decreased levels of testosterone, which are similar to the phenotypes of LGR4-hypomorphic mice. Tissue distribution of LGR4 was extensively analyzed in the mouse using gene-trap reporter enzyme alleles. However, its expression pattern in human tissues remained largely unknown. We have developed LGR4-specific monoclonal antibodies and used them to examine the expression of LGR4 in selected adult human and mouse tissues by immunohistochemical analysis. Intense LGR4-like immunoreactivity was observed in the epidermis and hair follicle of the skin, pancreatic islet cells, and epithelial cells in both the male and female reproductive organs. Of particular interest is that LGR4 is highly expressed in germ cells and pancreatic islet cells, which have important implications given the role of R-spondin-LGR4 signaling in the survival of adult stem cells. In addition, the majority of colon tumors showed elevated levels of LGR4 receptor. Overall, the expression pattern of LGR4 in human tissues mapped by this IHC analysis is similar to that in the mouse as revealed from gene trap alleles. Importantly, the pattern lends strong support to the important role of LGR4 in the development and maintenance of skin, kidney, reproductive systems, and other organs.

## Introduction

LGR4 (Leucine-rich repeat containing G protein-coupled Receptor 4), also known as GPR48, plays a pivotal role in development and potentially in colon carcinogenesis. Complete knockout of LGR4 led to total embryonic/neonatal lethality while hypomorphs had reduced viability with abnormalities in multiple organs, including the kidney [1,2], testis [3,4], skin [5], ovary [6], eye [7], mammary gland [8], intestine [9], and gall bladder [10]. A common theme found in the affected tissues with tubular structures is decreased proliferation of epithelial cells accompanied by reduced ductal branching and elongation. Just recently, a nonsense mutation of LGR4 was found to be associated with several human diseases, including reduced birth weights, electrolyte imbalance, and decreased levels of testosterone [11]. The remarkable phenotype overlaps between LGR4 knockout/hypomorphic mice and nonsense heterozygous humans indicate a highly conserved pathway implicated in multiple normal and patho-physiological processes [11]. 

LGR4 is also strongly implicated in the survival of stem cells in the gut and in colon cancer development. LGR5, a close homolog of LGR4, marks the rapidly cycling crypt stem cells [12]. However, it is LGR4 that is required for the self-renewal of this class of stem cells [9,13]. LGR4, LGR5, and the other closely related homolog LGR6 function as receptors of the R-spondin (RSPO) group of stem cell factors to potentiate Wnt signaling [13-15]. The four RSPO members (RSPO1-4) can all bind to and activate LGR4-6 with high affinity and potency to enhance Wnt-stimulated β-catenin signaling [13-16]. Wnt signaling is critical for normal development and the survival of adult stem cells in multiple tissues [17,18]. It is also aberrantly activated in ~90% of colon cancer through mutations in Apc and β-catenin [19]. Remarkably, recurrent, gain-of-expression gene fusion of RSPO2 (to EIF3E) and RSPO3 (PTPRK) were found in ~10% of human colon cancers [20]. Furthermore, the RSPO fusions were mutually exclusive with Apc and β-catenin mutations, suggesting that overexpression of RSPO2/3 had a driving role in tumorigenesis in the affected tumors [20]. Interestingly, only LGR4 was expressed at high levels in all tumors with the RSPO fusions, and could thus be the underlying receptor mediating the oncogenic effects of aberrant RSPO expression. 

Despite the wide range of critical roles of LGR4 in development and tumorigenesis, cellular distribution of the receptor in human tissues has not been determined, largely due to the lack of specific, sensitivity antibodies. Through gene reporter assays in transgenic mice, LGR4 was shown to be expressed in many epithelial tissues with particularly high levels in the kidney, adrenal gland, and testis in adult mice [21]. In LGR4-positive tissues, the receptor was generally expressed in proliferating stem cells and early progenitor cells [22]. Among human tissues, LGR4 mRNA was detected at significant levels in the pancreas, kidney, placenta, heart, ovary, testis, prostate, spleen, adrenal gland, trachea, spinal cord, thyroid, and stomach [1]. Here, we report the characterization of a panel of monoclonal LGR4 antibodies and the results of IHC analysis in selected normal and tumor tissues using LGR4-specific antibodies.

## Materials and Methods

### Antibody generation, purification, and characterization

 Monoclonal antibodies against human LGR4 were custom-generated at Aldevron (http://www.aldevron.com/antibody) using DNA-based immunization. Animals were housed at Janvier Labs in France and animal related protocols are approved by Ethics Committee of Janvier Labs. Animals were euthanised with carbon dioxide. Rats were immunized with full-length human LGR4 in an expression vector, hybridoma clones were created, and LGR4-immunoreactive clones were identified by FACS analysis using LGR4-expressing cells at Aldevron. Positive clones were analyzed and confirmed by immunocytochemistry and Western blot using HEK293T cells expressing human or mouse LGR4. Selected clones were cultured in serum-free media and antibodies were purified using protein G-based affinity purification following the manufacturer’s protocol. Purity of antibodies was confirmed by gel analysis. 

### Knockdown of LGR4 in cancer cell lines and Western blot analysis

Colon cancer cell lines HCT116 and HT29 were cultured in DMEM with 10% FBS + 100U/ml penicillin and 100 µg/ml streptomycin. To knockdown LGR4 in HT29 cells, the empty lentiviral vector pLKO and expression cassettes encoding LGR4-shRNA (5'-GCG TAA TCA AAT CTA CCA AAT-3') were purchased from Open Biosystem. Vector pLKO or shRNA construct, the packaging construct psPAX, and the envelope construct pMD2G were co-transfected at a ratio of 4:3:1 into HEK293T cells with FuGene HD (Roche). The conditioned media containing viral particles were collected after 24 and 48 hrs, pooled, and used for infection. HT29 cells were infected at 80% confluence with a viral suspension diluted with growth media overnight. Then, the media was changed to fresh growth media for 24 hrs. Subsequently, puromycin (4µg/ml final concentration) was added for selection and stable knockdown pools were obtained. For Western blot analysis, the cells were lysed in ice-cold RIPA lysis buffer (AMRESCO, N653) containing protease inhibitors (Roche, 11697498001). The cell debris was pelleted by centrifugation at 16,000g for 10 min. The supernatants were mixed with 5× SDS loading buffer and incubated at 37°C for 1 hr. The samples were analyzed by SDS-PAGE. LGR4 antibodies were used at 20 µg/ml final concentration. 

Plasmids containing full length mouse LGR4, human LGR4, LGR5 and LGR6 tagged with HA, Myc, and FLAG, respectively, were described before [14,23]. 293T cells were transiently transfected with each LGR plasmid, and cells were harvested 24 hrs later in RIPA lysis buffer. Recombinant LGRs were analyzed by western blot using anit-HA antibody (cell signaling, 3724), anti-myc antibody (cell signaling, 2278) and anti-FLAG antibody (Sigma, F4049). The two commercial LGR4 antibodies used for comparison were purchased from Rockland (200-301-B45S) and Santa Cruz (sc-68578). 

### Immunohistochemistry and Immunofluorescence

This study was carried out in strict accordance with the recommendations in the Institutional Animal Care and Use Committee (IACUC) for the University of Texas Health Science Center at Houston (UTHealth). The protocol was approved by the Animal Welfare Committee (Permit Number: 12-078). All efforts were made to minimize suffering. Mouse tissue sections from wild-type C57BL/6N mice used for this study were provided by Dr. Qingchun Tong at the Institute of Molecular Medicine, University of Texas Health Science Center at Houston. Microarrays of normal adult human tissues were purchased from US Biomax (Maryland, USA). Frozen colon tumor tissues were fixed and sectioned following standard procedures. The M.D. Anderson Cancer Center (MDACC) Institutional Review Board approved human specimen procurement, and informed consents were obtained. Prior to immunostaining, sections were deparaffinized in xylene for 15 min and rehydrated in a descending alcohol series. Antigen retrieval was carried out by boiling the slides in citrate buffer (pH 6.0) for 20 min. Subsequently, slides were incubated overnight with primary LGR4 monoclonal antibodies (5A3 for mouse LGR4 and 7E7 for human LGR4, 20 µg/ml) at 4°C The anti-rat Vectastain ABC system (Vector Laboratories, PK-6104) was used as secondary antibody and enhanced metal DAB (Thermo Scientific, #34065) was used as substrate. Counterstaining was performed with hematoxylin (Vector Laboratories, H-3404). The slides were mounted with permanent mounting media (Vector Laboratories, H-5000). Normal Rat IgG served as negative control. For normal human tissue sections, a minimum of three samples from three individuals were stained and similar results were obtained across the samples.

For immunofluorescence, paraffin sections were rehydrated following the protocol described above, and co-stained with LGR4 monoclonal antibody 5A3 (20 µg/ml) and insulin antibody (Santa Cruz, sc-9168). Sections were counterstained with TO-PRO-3. Images were recorded and analyzed using confocal laser scanning microscope (Leica; TCS SP5 microscope) with LAS AF Lite software.

## Results

### Characterization and Validation of LGR4-specific antibodies

**Figure 1:**
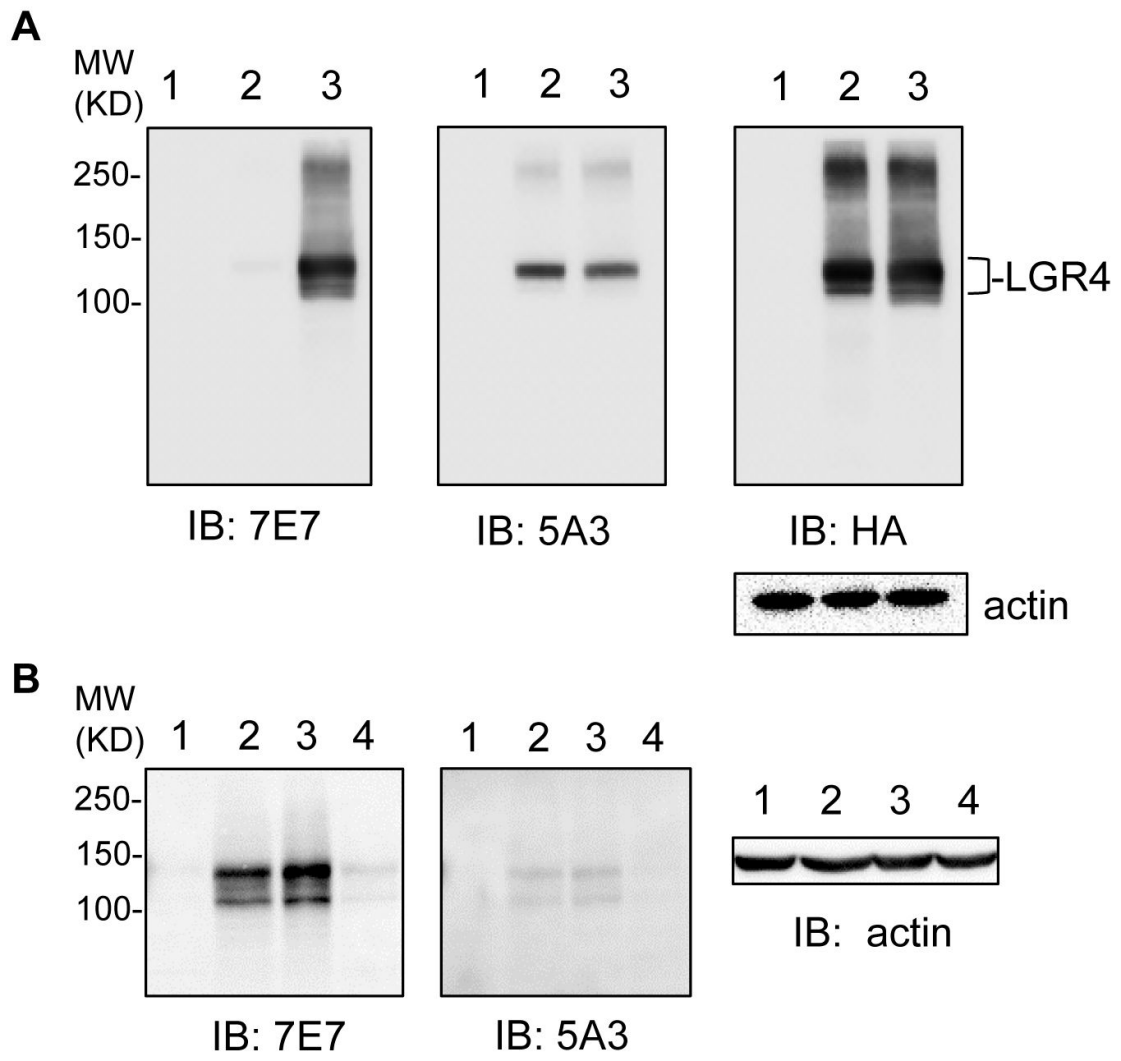
Recombinant and endogenous LGR4 were detected with custom generated rat monoclonal antibodies. A, Western blot of HEK293T transfected with vector (1), mouse LGR4 tagged with HA (2), or human LGR4 tagged with HA (3) using antibodies 7E7 (left panel), 5A3 (mid panel), and anti-HA (right panel). B, Western blot of LGR4 using 7E7 (left panel) and 5A3 (mid panel) on human colon tumor cell lines HCT116 (1), parental HT29 (2), vector-infected HT29 (3) and LGR4-shRNA infected HT29 (4). Actin was used as loading control.

DNA-based immunization using a vector expressing full-length human LGR4 was utilized to generate anti-LGR4 antibodies in the rat. Hybridoma clones were initially screened with cells expressing human LGR4 by FACS analysis. A panel of 13 clones showed reproducible binding to cells expressing LGR4 but not to vector control cells and they were subcloned and further characterized. We first screened supernatants of the hybridoma cultures by Western blot analysis on HEK293 cells expressing vector, mouse or human LGR4 with an HA tag. Clone 7E7 was found to detect human, but not mouse LGR4 with the correct size (Figure 1A, left panel) while clone 5A3 detected both human and mouse LGR4 with similar intensity (Figure 1A, mid panel). The identity of the signals were further confirmed with anti-HA antibody (Figure 1A, right panel). The bands at higher molecular weights (~250 KD) most likely represented dimers or oligomers of LGR4 as they were specifically detected by all three antibodies in LGR4-transfected cells, but not in vector control cells. These results indicate that both 7E7 and 5A3 can detect human LGR4 specifically with 5A3 being also capable of binding to mouse LGR4. 

We also tested 7E7 on recombinant LGR5 and LGR6 by immunoblotting and saw no cross-reactivity (Figure S1A). The expression and identity of all three LGRs were confirmed with antibodies against their respective tags (Figure S1A). Meanwhile, we tested two commercial antibodies of LGR4 on recombinant human LGR4. Neither one was able to detect LGR4 (Figure S1B).

Next, the antibodies were further analyzed on endogenous LGR4 using colon cancer cell lines. We have previously shown by qPCR that colon cancer cell line HT29 cells expressed moderate levels of LGR4 while HCT116 had negligible amounts [23], which is also consistent with the microarray expression data in CCLE (Cancer Cell Line Encyclopedia) [24]. Western blot with both 7E7 and 5A3 showed two bands between 110-130 KD in HT29 cells in a pattern similar to those from HEK293T cells expressing LGR4 (Figure 1B, Lane 2 of left and mid panels). In contrast, no specific band was observed with HCT116 cells (Figure 1B, Lane 1 of left and middle panels). HT29 cells expressing an LGR4 shRNA or vector control were also generated. The intensity of the bands from both 7E7 and 5A3 was significantly reduced in cells expressing LGR4-shRNA when compared to vector cells (Figure 1B, Lane 3 and 4 of left and mid panels). In addition, no other proteins were detected with significant intensity (Figure 1). Overall, these analyses indicate that 7E7 can specifically detect denatured human LGR4 while 5A3 reacts with both the human and mouse receptor with slightly lower sensitivity. As the distribution of LGR4 transcript in the mouse has been extensively analyzed using reporter enzyme alleles, we undertook the task of mapping the location of LGR4 in human tissues, particular in those affected by LGR4 deficiency, including the skin, testis, ovary, uterus, mammary gland, intestine, and the pancreas.

### Expression of LGR4 in the skin

**Figure 2:**
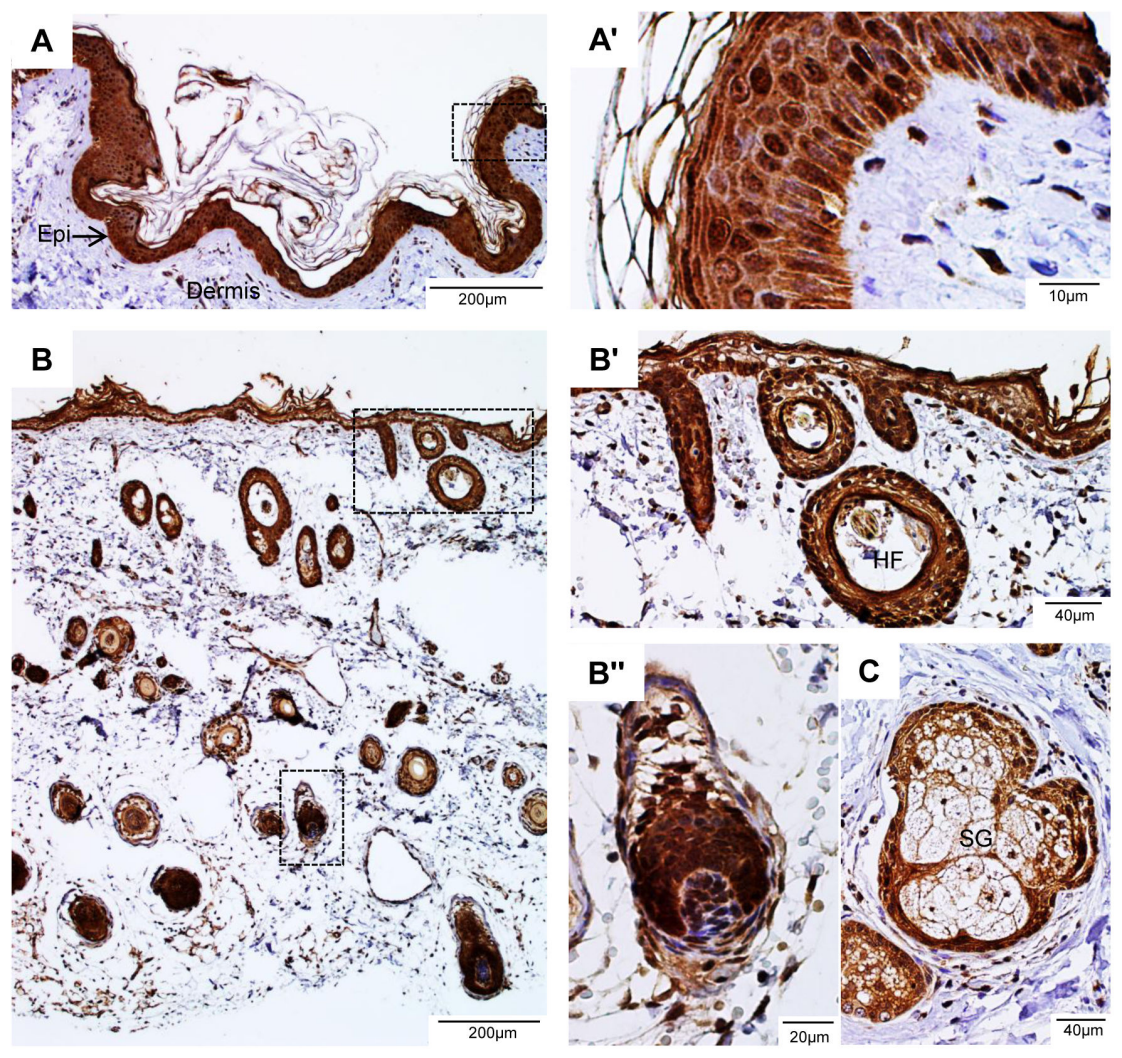
LGR4 is highly expressed in the epidermis and hair follicles of human skin. A, representative micrograph of a skin section stained with 7E7, Epi – epidermis. The boxed area is enlarged in A'. B, lower magnification of a skin section showing staining of hair follicles. The boxed areas are enlarged in B' and B'', HF – hair follicle. C, staining was shown in the basal layer of sebaceous gland (SG).

Staining with 7E7 revealed that strong LGR4-like immunoreactivity (IR) was found in the granular, spinous, and basal/germinal layers of the skin (Figure 2A-B). More intense staining was seen in the granular layer and some cells of the basal layer (Figure 2A'). Staining was not observed in the cornified and translucent layers of the epidermis or in the dermis. The strong expression of LGR4 in basal and granular cells is consistent with the findings that LGR4 plays a critical role in these cells as loss of LGR4 or RSPO1 led to increased risk of squamous cell carcinoma of the skin in human [11,25]. 

In the hair, strong LGR4-IR was seen in the external root sheath (Figure 2B-B'') and the basal layer of sebaceous gland (Figure 2C). No hair defect, intriguingly, was reported in human subjects with LGR4 haplotype insufficiency or recessive loss of RSPO1. However, conditional complete knockout of LGR4 in mouse skin led to partial impairment of hair follicle development [5]. Interestingly, in the skin, LGR5 and LGR6 are markers of distinct populations of stem cells while LGR4 is expressed in both types of stem cells [26]. As loss of LGR5 or LGR6 had no effect on skin development, these data indicate that LGR4 is the predominant receptor mediating the activity of RSPOs in the skin.

### Expression of LGR4 in the reproductive system

**Figure 3:**
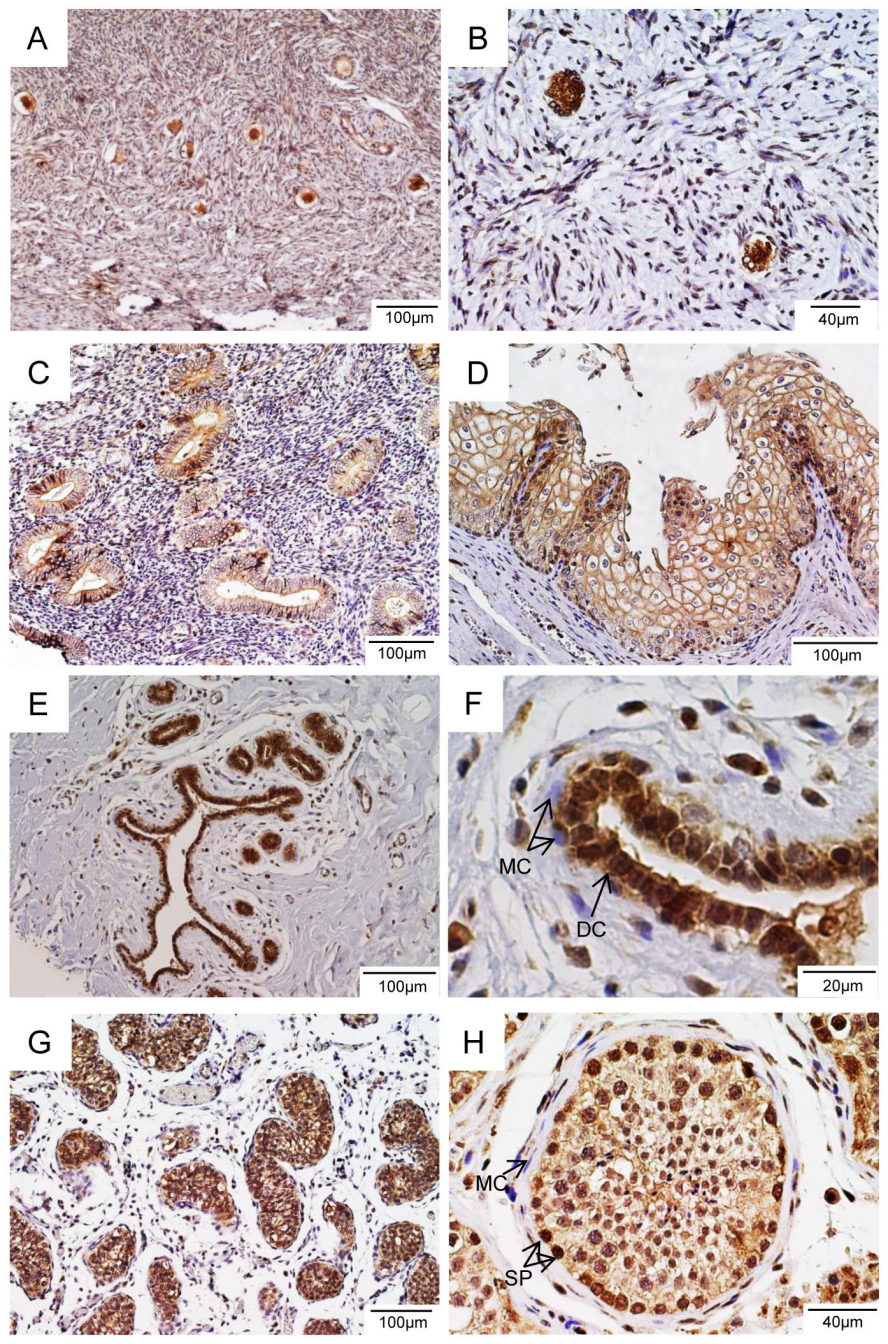
LGR4 is highly expressed in germ cells and epithelial cells in human reproductive organs. A, ovary, B, primordial follicles of ovary, C, uterine glands, D, stratified squamous epithelium of uterine cervix, E, mammary duct, F, breast intralobular terminal duct, DC – duct cells, MC – myoepithelial cells, G, testis, H, seminiferous tube of testis, SP – spermatogonia, MC – myoid cells.

Knockout of LGR4 impairs the development of both male and female reproductive systems in mice [3,4,6,8]. Deficiency of RSPO1 in humans leads to defective development of the ovary and thus female-to-male sex reversal [25], whereas carriers of an LGR4 nonsense mutation had delayed menarches and reduced testosterone levels [11]. We analyzed expression of LGR4 in selected tissues of the human reproductive system with 7E7. In the ovary, intense LGR4-IR was only found in promordial and primary follicles (Figure 3A-B). Interestingly, RSPO2 was recently reported to stimulate the development of primary follicles in the mouse [27]. These data suggest that RSPO2-LGR4 signaling may have a similar function in human follicle development. In the uterus, moderate levels of LGR4-IR were observed in the glandular epithelial tissues (Figure 3C) and the basal layer of the uterine surface (Figure 3D). Specific roles of LGR4 in uterus have yet to be revealed. In the breast, intense signal of LGR4-IR was observed in the mammary ducts (Figure 3E), largely in the duct epithelial cells, but not in myoepithelial cells (Figure 3F). In the mouse, high levels of LGR4 promoter activity was also found in the ductal epithelial cells [8] as well in basal cells [28], and knockout of LGR4 or RSPO1 led to reduction in ductal elongation, branching and the number of terminal buds [8,28]. The intense staining of LGR4 in ductal epithelial cells of human breast suggests that this receptor may also have a critical role in the branching and elongation of mammary ducts in women. In the testis, a strong signal of LGR4-IR was seen in the seminiferous tube (Figure 3G), mostly in the spermatogonia, but not in the peritubular myoid cells (Figure 3H). Intriguingly, Qian et al. recently reported that murine LGR4 was expressed only in myoid cells in the testis [29], suggesting that there is a major difference in the location of LGR4 expression in the testis between human and mouse. No significant expression of LGR4 was found in the prostate (Figure S2). Overall, these IHC results indicate that human LGR4 is expressed in a pattern very similar to that of the mouse in adult tissues of the male and female reproductive systems, except in the testis. Furthermore, the pattern of expression supports a critical role of LGR4 in reproductive functions in human [11].

### Expression of LGR4 in normal intestine and colon cancer

**Figure 4:**
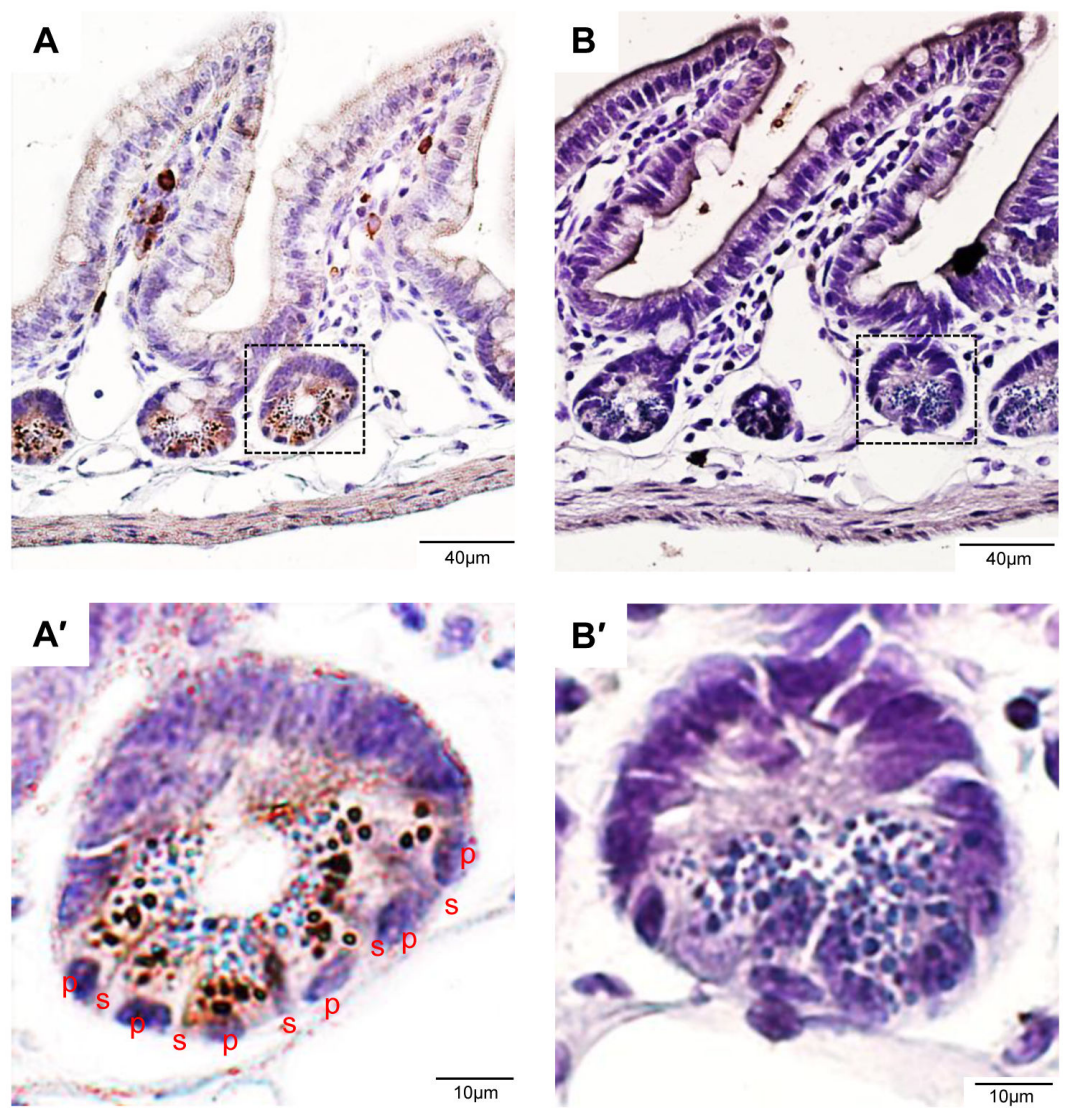
LGR4 is strongly expressed in the Paneth cells in mouse intestine. A, an ileum section stained with the antibody 5A3. B, an adjacent section stained with normal rat IgG. A' and B', enlarged view of the boxed area in A and B, respectively. P – Paneth cells, s – stem cells.

Using the antibody 5A3, we first examined the distribution of LGR4 in murine intestine where its pattern of transcription had been analyzed in detail by several groups [9,13,30]. After staining of ileum sections with 5A3, intense LGR4-IR in a vesicular pattern was observed in Paneth cells which were distinguished by large secretory granules in the cytoplasm (Figure 4A). Similar staining was also noted in crypt stem cells which are sandwiched between Paneth cells [31] (Figure 4A'). The vesicular pattern of LGR4-IR is most likely due to strong internalization of this receptor as we reported previously [14]. No strong staining of LGR4 was found beyond the cells in the crypts. In contrast, control rat IgG only gave strong staining in discrete cells in intervillus region (Figure 4B-B'), which were most likely plasma cells that can give strong, non-specific staining due to high levels of intracellular IgG. The intense LGR4-IR signal observed in Paneth cells and stem cells in the crypt bottom of mouse intestine were consistent with that of LGR4 mRNA distribution determined using lac-Z alleles [13,30]. Therefore, the results confirm high receptor levels of LGR4 in these cells and validate the specificity of the antibody.

In the mouse colon, only diffuse, weak LGR4-IR in the cytoplasm was found in all cells from the crypt bottom to the epithelial surface, with slightly stronger staining at the surface (Figure S3). Surprisingly, no distinct staining was found in stem cells located at the crypt bottom (Figure S3). Control rat IgG again only gave intense staining in cells located between crypts (most likely plasma cells) (Figure S3). Interestingly, LGR4 promoter activity in the colon as detected by lacZ staining was observed in the bottom half of the crypts [9,30], which appeared to be weaker than that in the crypts of the small intestine [30]. Our finding of weak LGR4-IR in the mouse colon epithelium also confirms that LGR4 levels are lower in the colon which does not have Paneth cells. 

We then used the antibody 7E7 to determine expression of LGR4 in human small intestine. Only weak, diffuse staining was observed in epithelial cells (Figure S4A). In the bottom of the crypts, LGR4-IR appeared to be stronger in the stem cells compared to the Paneth cells (Figure S4A'). In contrast to the staining in the small intestine of the mouse, no intense, vesicular staining was observed in the Paneth cells in human small intestine (Figure S4A-A'). In human colon, little to no LGR4-IR was found in all epithelial cells (Figure S4B). These results indicate that in human, expression of LGR4 in the intestine was less abundant than in the skin or reproductive tissues, despite the fact that LGR4 is essential for the survival of intestinal stem cells in the mouse [9,13]. 

**Figure 5:**
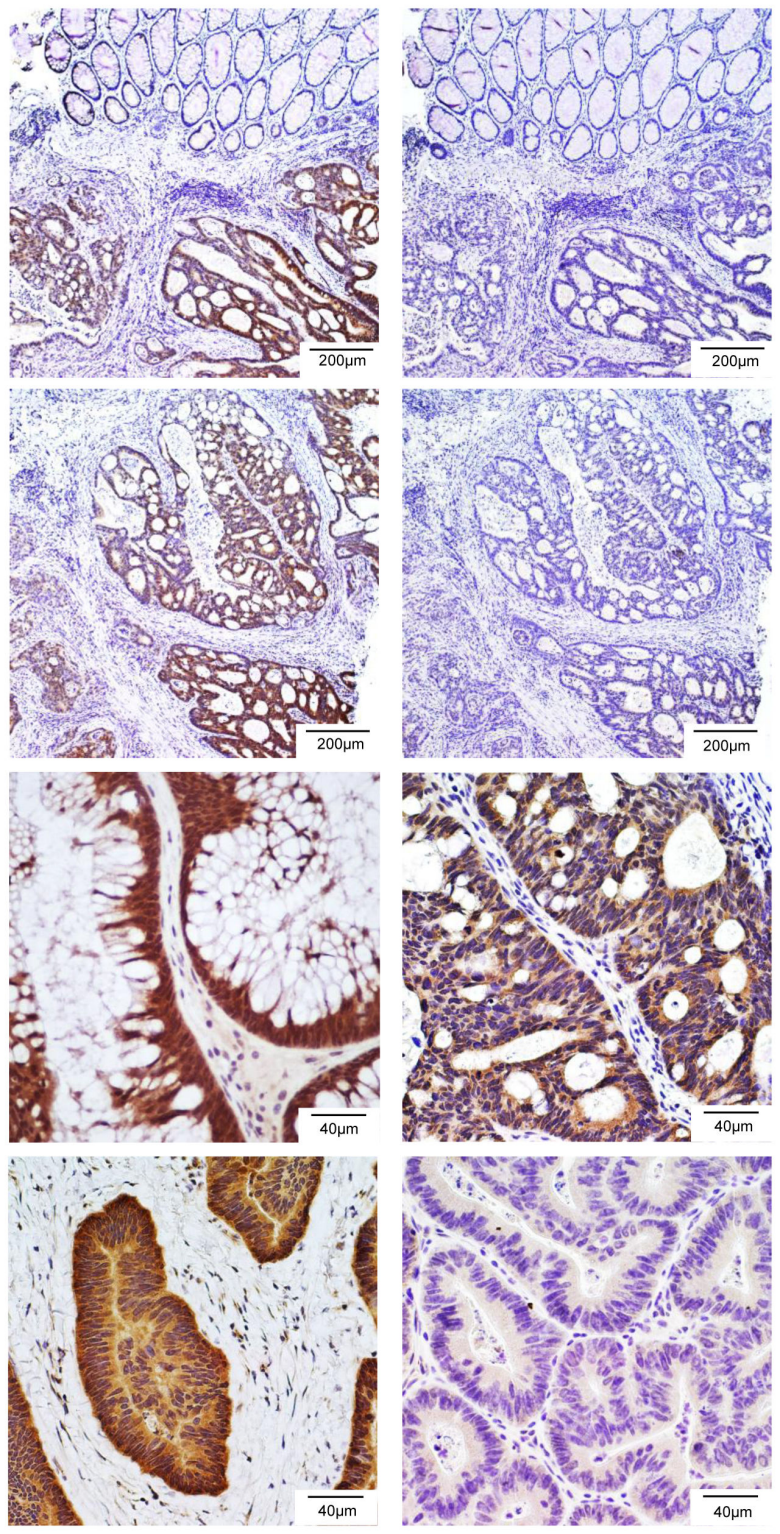
LGR4 is overexpressed in a significant fraction of human colon cancers. A and C, Staining of two colon tumor samples with 7E7. Specific and strong staining on colon tumor epithelial layers was clearly notable. B and D, Staining of adjacent sections of A and C, respectively, with 7E7 preincubated with human LGR4-ECD. The signal was blocked by incubation with LGR4-ECD. E-G, Three representatives of colon tumors with strong LGR4-IR. H, One representative of colon tumors with no expression of LGR4.

Next, we examined the expression of LGR4 in human colon cancers and their normal adjacent tissues with 7E7 given the finding of recurrent RSPO2/3 gene fusions in human colon cancer [20]. Strong LGR4-IR was observed in some colon tumors as shown by the representative samples (Figure 5 A, C, E-G). Increased staining in tumor vs normal crypts was clearly visible in Sample A. Furthermore, the staining was completely competed off in the presence of excess amounts of LGR4-ECD, providing additional evidence that the antibody is specific to LGR4 (Figure 5B & D). We stained a total of 33 human colon tumor samples with 7E7 and found that 67% (22/33) were moderately to strongly positive on LGR4, three of which are shown in Figure 5E-G. The intensity of LGR4-IR was largely uniform across different regions of each tumor, implying a lack of intratumoral heterogeneity in LGR4 expression. Approximately 15% (5/33) were nearly negative of LGR4-IR with one representative shown (Figure 4H). Overall, these results indicate that LGR4 expression was increased in the majority of colon cancers, consistent with previous findings based on mRNA analysis [32]. 

### Expression of LGR4 in other tissues

**Figure 6:**
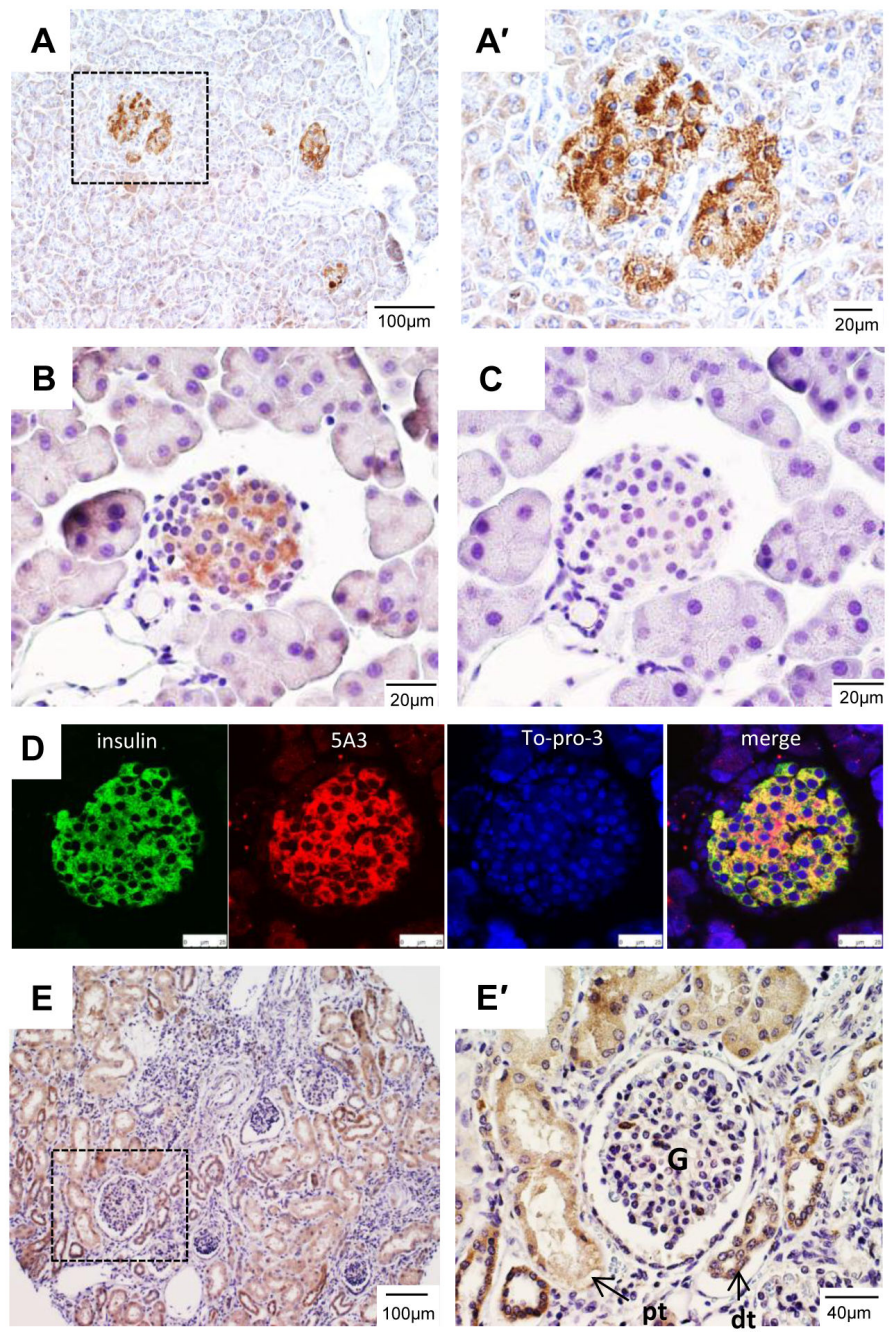
Expression of LGR4 in the pancreas and kidney. A, Staining of human pancreas with 7E7. A', Enlarged view of the area boxed in A. B, Staining of mouse pancreas with 5A3. C, Staining of an adjacent section of B with normal rat IgG as negative control. D, Co-staining of mouse pancreas with 5A3 and insulin antibody. E, Staining of human kidney, E', an enlarged view of the boxed area in E. G – glomerulus, pt – proximal convoluted tubule, dt – distal convoluted tubule.

Intense LGR4-IR was found in the pancreatic islets except in the surrounding myoepithelial cells (Figure 6A) with no staining in acinar cells (Figure 6A'). A similar pattern was also found in mouse pancreas (Figure 6B). Control rat IgG gave no signal (Figure 6C). Moreover, co-staining of 5A3 and an anti-insulin antibody on mouse pancreas sections showed that LGR4 was expressed in all beta cells (Figure 6D). Previously, it was shown that RSPO1 stimulated insulin secretion and beta-cell growth through potentiation of Wnt signaling [33]. More recently, it was reported that R-spondin and Wnt increased the proliferative potential of adult human beta-cells without disrupting their differentiated phenotype [34]. However, the underlying receptors for R-spondin activity in beta-cells were not characterized. Of the three LGR receptors, only LGR4 was expressed in the pancreas based on the analysis of EST data and Northern blot [35]. The intense staining of LGR4 in the islets strongly suggests that LGR4 mediates the effects of RSPOs in the pancreas. In the kidney, moderate LGR4-IR was found in both the proximal and distal tubules, but not in the glomeruli (Figure 6E-E'), similar to the pattern in the mouse kidney [21]. As carriers of an LGR4 nonsense mutation showed electrolyte imbalance in a similar fashion to that of LGR4-hypomorphic mice [11,36], the moderate staining of LGR4 in proximal and distal tubules of human kidney suggests that LGR4 function is important to the maintenance of adult kidney epithelial cells. 

## Discussion

The recent finding that haplotype insufficiency of LGR4 is associated with several diseases indicates that LGR4 has a critical role in human development and physiological functions in adults [11]. The type and location of LGR4-expressing cells in human adult tissues, however, remained undetermined. We carried out IHC analysis across a panel of human adult tissues with a LGR4-specific monoclonal antibody and found that its expression pattern in human is largely similar to that in the mouse, which is consistent with phenotypic overlaps between haplotype insufficient humans and hypomorphic mice [11]. A major developmental defect in LGR4 knockout or hypomorphic mice is reduced tubule elongation and branching in several tissues with tubular structures, including the kidney, testis, and mammary gland. Strong staining of LGR4 was found in epithelial cells in these tissues of adult humans, suggesting that LGR4 still plays a role in the maintenance of tissue functions. On the other hand, no staining of LGR4 was found in epithelial cells of some tissues with tubular structures, such as the pancreas, liver and lung which were not affected in LGR4 knockout or hypomorphic mouse. 

The highest observed receptor level of LGR4 was found in the epidermis of the skin with intense staining in selected cells in the basal layer and granular layers. Loss of either RSPO1 or LGR4 increases the risk of developing squamous cell carcinoma of the skin, indicating that RSPO1-LGR4 signaling plays a vital role in the negative regulation of cell proliferation in the skin. RSPO1 stimulation of LGR4 potentiates Wnt signaling which is generally oncogenic [13-15,37], and overexpression of RSPO2 and RSPO3 is associated with carcinogenesis in human colon cancer as well as in mouse models of breast and colon cancer [20,38-40]. LGR4 was clearly upregulated in a significant fraction of human colon cancer (Figure 5). It was also the only receptor that was consistently expressed in colon tumors with RSPO2 or RSPO3 gene fusions [20]. Therefore, the role of RSPO-LGR4 signaling in cancer development is context dependent, similar to that of Notch in solid tumors [41]. 

In summary, LGR4 is expressed abundantly in epithelial cells of selected tissues in adult humans. In particular, high levels of LGR4 receptor were found in basal and granular cells in the skin, ductal epithelial cells in the breast, spermatogonia and tubule cells in the testis, islet cells in the pancreas, and tumor cells in the majority of colon cancer cases. Overall, the expression pattern of LGR4 in adult human tissues strongly supports its role in the physiological functions of kidney, testis, and breast, and potentially in the development of some colon cancers. 

## Supporting Information

Figure S1 **Western Blot analysis of recombinant human LGR4, LGR5 and LGR6 with 7E7 and two commercial antibodies.** Neither of the commercial antibodies is working.
(PDF)

Figure S2 **Staining of human prostate with 7E7.** No specific staining was observed.
(PDF)

Figure S3 **Staining of mouse colon with 5A3 (left panel) and normal rat IgG (right panel).**
(PDF)

Figure S4 **Staining of human intestine with 7E7.** A, small intestine. A', Enlarged view of the boxed area in A, p – paneth cells, s – stem cells. B, colon.
(PDF)
