# Supplementary material for: Analysis of LGR4 Receptor Distribution in Human and Mouse Tissues
Source: PLoS One. 2013 Oct 21;8(10):e78144. doi: 10.1371/journal.pone.0078144 (PMC3804454; doi:10.1371/journal.pone.0078144)
Supplement: Figure S1 — Western Blot analysis of recombinant human LGR4, LGR5 and LGR6 with 7E7 and two commercial antibodies. Neither of the commercial antibodies is working. (PDF) [file pone.0078144.s001.pdf]

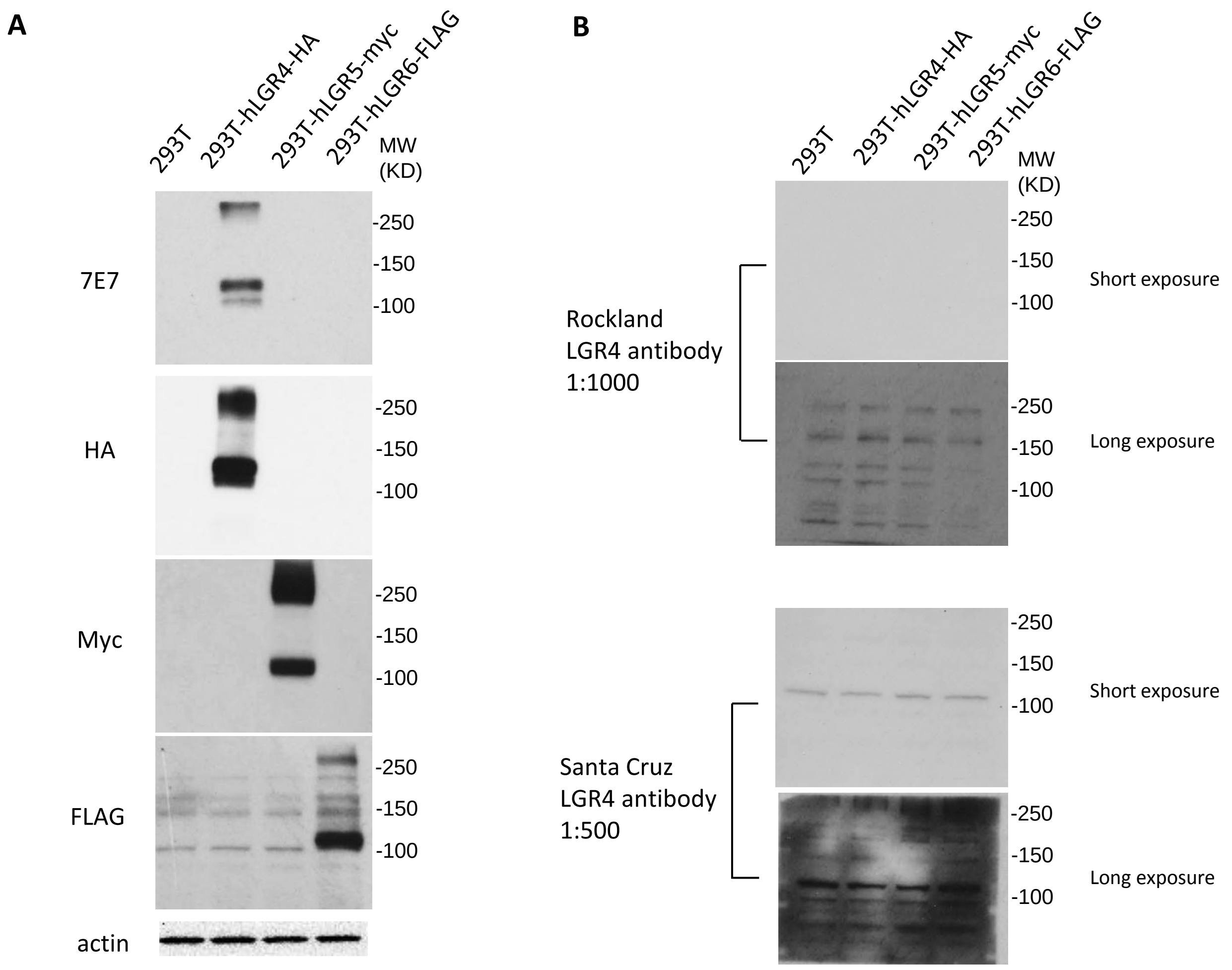

Figure S1. Western Blot analysis of recombinant human LGR4, LGR5 and LGR6 with 7E7 and two commercial antibodies. Neitherof the commercial antibodies is working.
