# Supplementary figures and images for: Analysis of LGR4 Receptor Distribution in Human and Mouse Tissues
Source: PLoS One. 2013 Oct 21;8(10):e78144. doi: 10.1371/journal.pone.0078144 (PMC3804454; doi:10.1371/journal.pone.0078144)

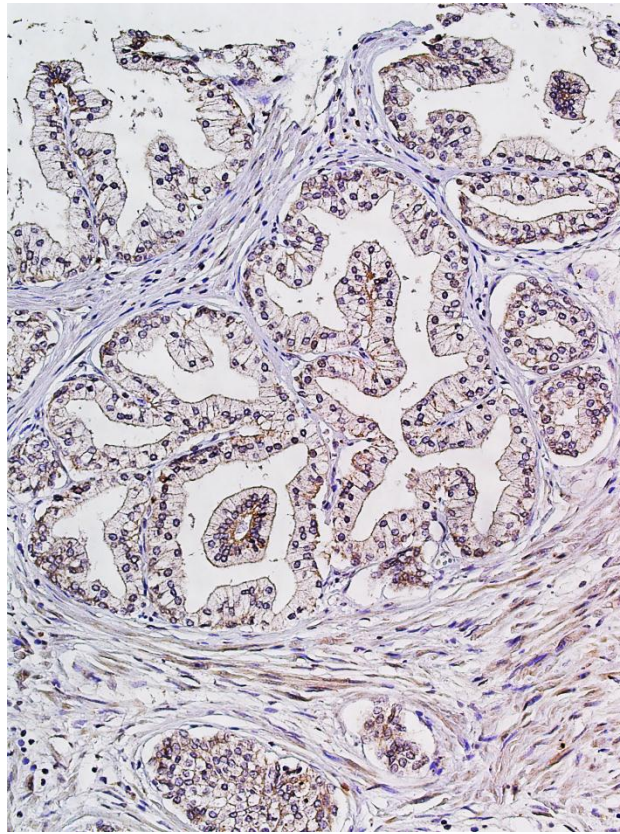

Figure S2. Staining of human prostate with 7E7. No specific staining was observed.

Supplement: Figure S2 — Staining of human prostate with 7E7. No specific staining was observed. (PDF) [file pone.0078144.s002.pdf]
