# Supplementary material for: Analysis of LGR4 Receptor Distribution in Human and Mouse Tissues
Source: PLoS One. 2013 Oct 21;8(10):e78144. doi: 10.1371/journal.pone.0078144 (PMC3804454; doi:10.1371/journal.pone.0078144)
Supplement: Figure S3 — Staining of mouse colon with 5A3 (left panel) and normal rat IgG (right panel). (PDF) [file pone.0078144.s003.pdf]

5A3 (anti-LGR4) Normal rat IgG

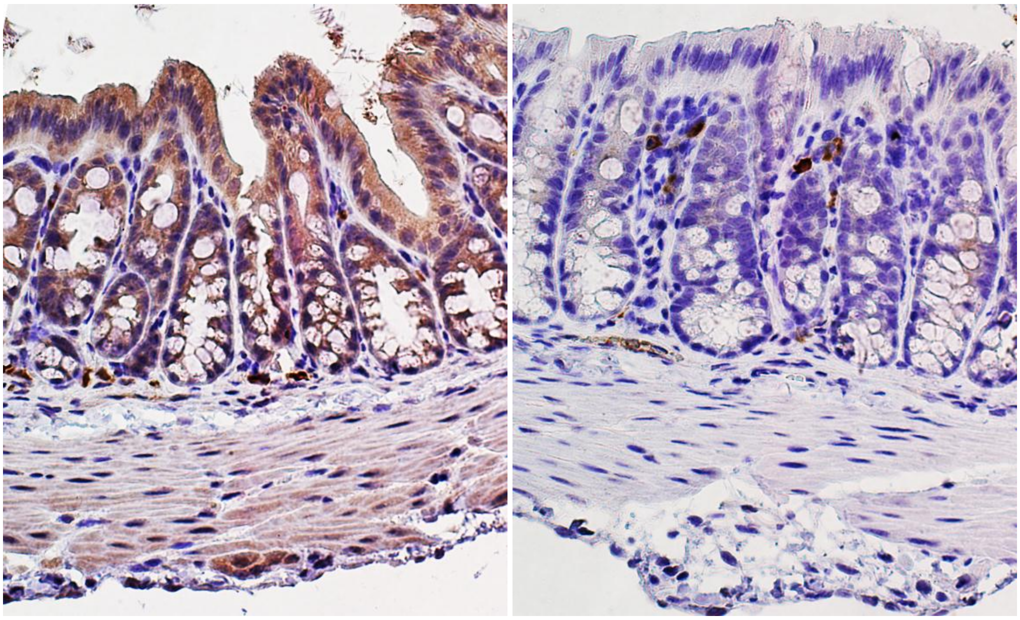

Figure S3. Staining of mouse colon with 5A3 (left panel) and normal rat IgG (right panel).
