# Supplementary material for: Analysis of LGR4 Receptor Distribution in Human and Mouse Tissues
Source: PLoS One. 2013 Oct 21;8(10):e78144. doi: 10.1371/journal.pone.0078144 (PMC3804454; doi:10.1371/journal.pone.0078144)
Supplement: Figure S4 — Staining of human intestine with 7E7. A, small intestine. A', Enlarged view of the boxed area in A, p – paneth cells, s – stem cells. B, colon. (PDF) [file pone.0078144.s004.pdf]

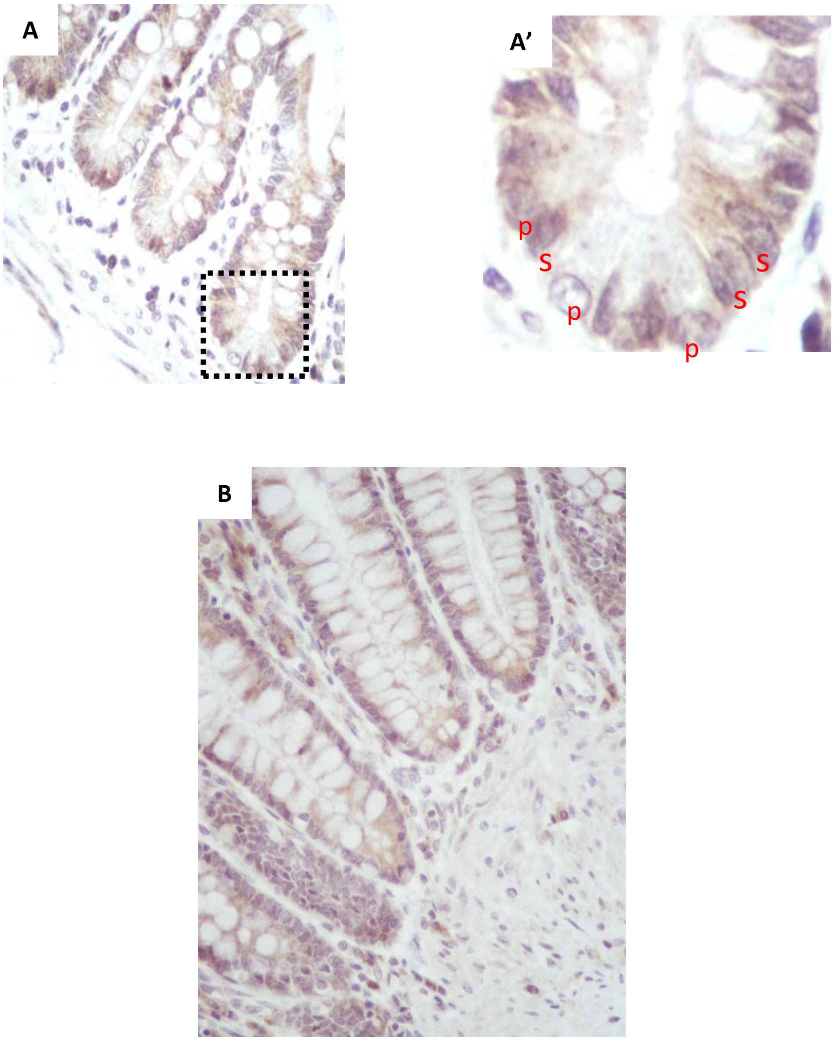

Figure S4. Staining of human intestine with 7E7. A, small intestine. A', Enlarged view of the boxed area in A, p – paneth cells, s – stem cells. B, colon.
